# Cutaneous Nodules and Inflammatory Arthritis: Two Illustrative Cases of Rare Mimics of Rheumatoid Arthritis

**DOI:** 10.3390/jcm14144940

**Published:** 2025-07-12

**Authors:** Reena Yaman, David J. DiCaudo, Olayemi Sokumbi, Michael M. Pham, Fawad Aslam, W. Leroy Griffing, Megan M. Sullivan

**Affiliations:** 1 Department of Rheumatology, Mayo Clinic, Jacksonville, FL 32224, USA; 2 Departments of Dermatology, Laboratory Medicine & Pathology, Mayo Clinic, Scottsdale, AZ 85259, USA; 3 Departments of Dermatology, Laboratory Medicine & Pathology, Mayo Clinic, Jacksonville, FL 32224, USA; 4 Department of Rheumatology, Mayo Clinic, Scottsdale, AZ 85259, USA

**Keywords:** arthritis, rheumatoid arthritis, fibroblastic rheumatism, multicentric reticulohistiocytosis, rheumatoid nodule

## Abstract

**Background:** Rheumatoid arthritis is a relatively common rheumatic disease that can present with inflammatory arthritis and subcutaneous nodules. Multicentric reticulohistiocytosis and fibroblastic rheumatism are rarer entities that also present with these features. **Methods:** Two cases, one of each of fibroblastic rheumatism and multicentric reticulohistiocytosis, are described highlighting characteristic clinical, radiographic, and histologic findings. A narrative review of the literature on these rarer conditions, compared with rheumatoid arthritis, is provided with a focus on articular and cutaneous findings, available information on disease presentations, and key contrasting features that can aid in diagnosis. **Results:** Radiographic erosion distribution and joint space narrowing, clinical nodule distribution and characteristics, and nodule histology can differ between these diseases. **Conclusions:** Multicentric reticulohistiocytosis and fibroblastic rheumatism should be considered in the evaluation of seronegative rheumatoid arthritis, especially in cases that do not respond predictably to standard therapies, and cutaneous nodule biopsy can aid in differentiating these three conditions.

## 1. Introduction

**Figure 1:**
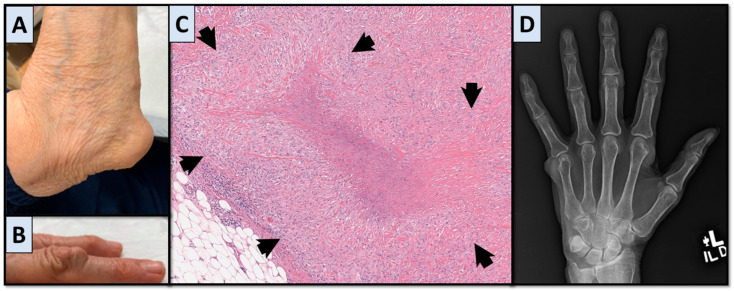
Rheumatoid arthritis. (**A**) Left elbow with large rheumatoid nodule. (**B**) Rheumatoid nodule overlying the 4th digit PIP (proximal interphalangeal) joint. (**C**) Rheumatoid nodule, left elbow biopsy showing palisading granuloma (arrows) with central fibrin (hematoxylin-eosin, original magnification ×6 by digital scan). (**D**) Left-hand radiograph demonstrating left 5th MCP (metacarpophalangeal) erosion and soft tissue nodule, mild radiocarpal and intercarpal joint space narrowing, 4th and 5th MCP joint erosions, 4th digit PIP erosion, 2nd digit MCP and 3rd digit PIP rheumatoid nodules.

Within the field of rheumatology, there are a myriad of conditions that can cause inflammatory arthritis. Rheumatoid arthritis (RA) is the most common, affecting an estimated 0.5–1% of the population [1]. Rheumatoid nodules are a common extra-articular disease manifestation with an estimated prevalence of 10% of those suffering from RA. Subcutaneous rheumatoid nodules can be located anywhere but are classically seen on extensor surfaces. Rheumatoid nodules can result in cosmetic and, less frequently, functional symptoms. Subcutaneous rheumatoid nodules are correlated with increased RA severity and seropositive disease. Histopathology typically demonstrates palisading granulomatous inflammation with a central region of fibrin; however, biopsy and histopathologic evaluation are infrequently necessary as rheumatoid nodules are usually asymptomatic and relatively common with classic presentations [2]. Characteristic findings are shown in Figure 1. Alternate diagnoses and nodule biopsy may be considered in patients with atypical presentation and seronegative disease. We describe two cases of rare conditions, fibroblastic rheumatism (FR) and multicentric reticulohistiocytosis (MRH), that may mimic RA and present with cutaneous nodules and inflammatory arthritis. 

## 2. Case 1

**Figure 2:**
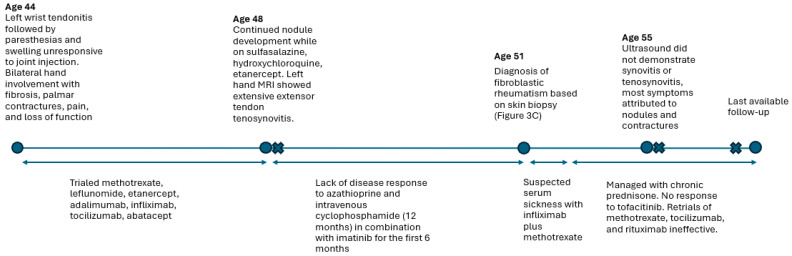
Case 1 timeline. Pertinent clinical results and findings shown by age. X-ray timepoints delineated by X. Trialed medications and medication periods shown with a double arrow line.

A 48-year-old female presented to rheumatology for evaluation of an unspecified arthropathy initially thought to be seronegative rheumatoid arthritis. This initially presented as tendonitis of the left wrist at age 44. This did not respond to injection and progressed to include paresthesia and swelling. She subsequently developed nodules associated with fibrosis and contractures on the bilateral hands, more frequently occurring on the palmar surface but also affecting the dorsal surface with associated pain, as well as loss of function and range of motion. Autoantibody testing, including rheumatoid factor, as well as antinuclear, cyclic citrullinated peptide, SS-A, SS-B, RNP, Smith, Scl 70, Jo1 antibodies, was negative, and inflammatory markers and creatine kinase remained within the normal range. At the time of presentation, she was on sulfasalazine, hydroxychloroquine, and etanercept and had previously trialed multiple conventional synthetic and biologic disease-modifying anti-rheumatic medications for treatment of presumed rheumatoid arthritis, including methotrexate, leflunomide, etanercept, infliximab, tocilizumab, and abatacept. Unexpectedly, hand X-rays showed progressive erosions 6 years following diagnosis of seronegative rheumatoid arthritis, as well as severe associated osteoarthritis. Left-hand MRI showed extensive tenosynovitis of the extensor tendons. Due to diagnostic uncertainty and atypical features for rheumatoid arthritis, including radiologic and cutaneous findings, as well as response to treatment, biopsies were pursued. Left wrist synovial biopsy demonstrated patchy villous architecture and mild reactive synovial hyperplasia without signs of inflammation. A punch biopsy of a representative nodule from the left middle finger flexor sheath and thumb demonstrated fibromatosis characterized by dense hyalinized collagen deposition and fascicles of parallel fibroblasts with bland nuclear features. She was diagnosed with FR by dermatology based on skin biopsy results 6 years following initial presentation. Over the course of her disease, she was ultimately trialed on azathioprine, cyclophosphamide (12-month intravenous course overlapping with imatinib for 6 months), tofacitinib, and rituximab, none of which provided benefit. Her second trial of infliximab was associated with suspected serum sickness. Given the lack of benefit to most other forms of immunosuppression, her disease was managed with prednisone 10–20 mg daily, which provided mild benefit. A timeline of Case 1 is shown in Figure 2.

**Figure 3:**
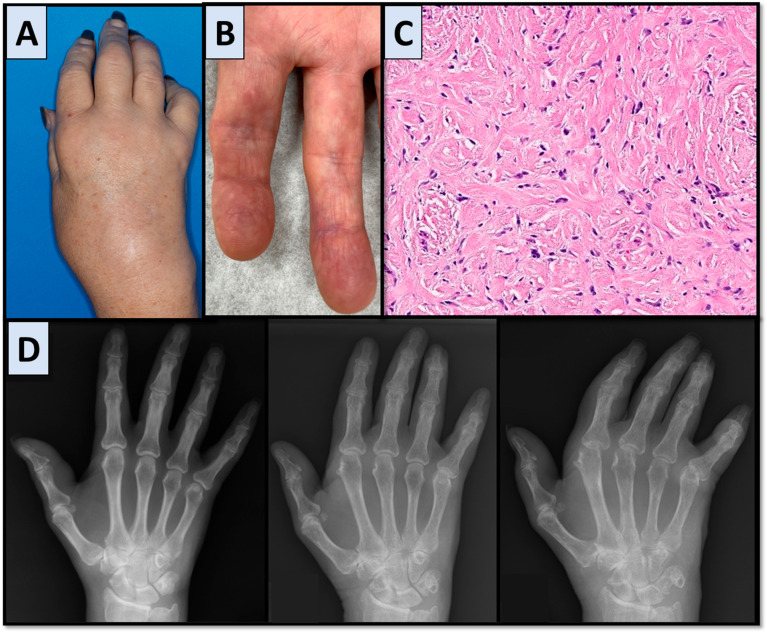
Fibroblastic rheumatism images. (**A**) Right-hand photograph from Case 1. (**B**) Photograph showing a flesh-colored, waxy nodule on the left 2nd digit over the volar surface from another patient with FR. (**C**) Right palm nodule biopsy showing increased fibroblasts and scattered binucleated and multinucleated giant cells within a sclerotic dermis from Case 1 (hematoxylin-eosin, original magnification ×16 by digital scan). (**D**) Right-hand X-rays showing progression over time from Case 1. Left to right: baseline, 7, and 9 years after symptom onset.

FR is a rare disease entity characterized by nontender cutaneous nodules and other rheumatologic findings, including polyarthritis and contractures. FR was first described by Chaouat et al. in 1980 [3]. Given its rarity, available information on this disease is based on published case reports. A recent systematic review of all published case reports and case series has summarized patient demographics and the prevalence of various disease characteristics [4]. This included 39 studies, 23 describing adult cases and 11 describing pediatric cases, 8 of which were only published in abstract form. FR demonstrates a male predominance with a male-to-female ratio of 2:1 in adults and 3:1 in children. Most patients presented with nontender nodules, arthralgias, morning stiffness, and finger contractures associated with erosive and destructive lesions seen on radiographic imaging. Patients had a median of 20 nodules with a median nodule diameter of 15 mm. Laboratory testing is generally unremarkable [5]. Histology of skin samples demonstrated thickened dermis containing collagen fibers with myofibroblasts [6]. Diagnostic or classification criteria do not exist, and there is currently no guideline-based treatment for this disease, with the most commonly used treatments including prednisone, methotrexate, and nonsteroidal anti-inflammatory drugs (NSAIDs). More than half of the cases showed only a partial response to therapy. Additionally, some authors report success with supportive and physical therapy alone [5]. Characteristic FR findings are shown in Figure 3.

## 3. Case 2

**Figure 4:**
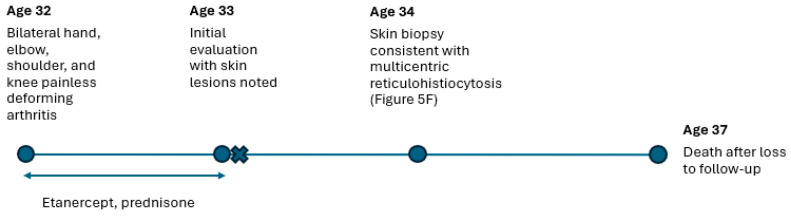
Case 2 timeline. Pertinent clinical results and findings shown by age. X-ray timepoints delineated by X. Trialed medications and medication periods shown with a double arrow line.

A 33-year-old male presented with a 1-year history of a disfiguring arthritis without pain. He had bilateral hand, elbow, shoulder, and knee involvement and, within several months of onset, was wheelchair-bound and had limited use of the hands. Flesh-colored papules and nodules were noted on the bilateral hands, elbows, upper shoulders, and ears. Laboratory evaluation demonstrated C-reactive protein (CRP) was elevated to 30.9 mg/L and erythrocyte sedimentation rate (ESR) was 21 mm/hr. Rheumatoid factor, as well as anti-cyclic citrullinated peptide, nuclear, B2 glycoprotein IgG/IgM, phospholipid IgG/IgM, SS-A, SS-B, Smith, SNP, Scl 70, Jo 1 antibodies were negative. He had been treated with intermittent courses of prednisone followed by the initiation of etanercept. Skin biopsy was recommended for diagnosis confirmation. Punch biopsy of a nodule from the left neck demonstrated diffuse dermal infiltration of large mononuclear and multinucleated histiocytes with abundant pink cytoplasm characteristic of MRH. Unfortunately, the patient was lost to follow-up and died within 4 years of evaluation. A timeline of Case 2 is shown in Figure 4.

**Figure 5:**
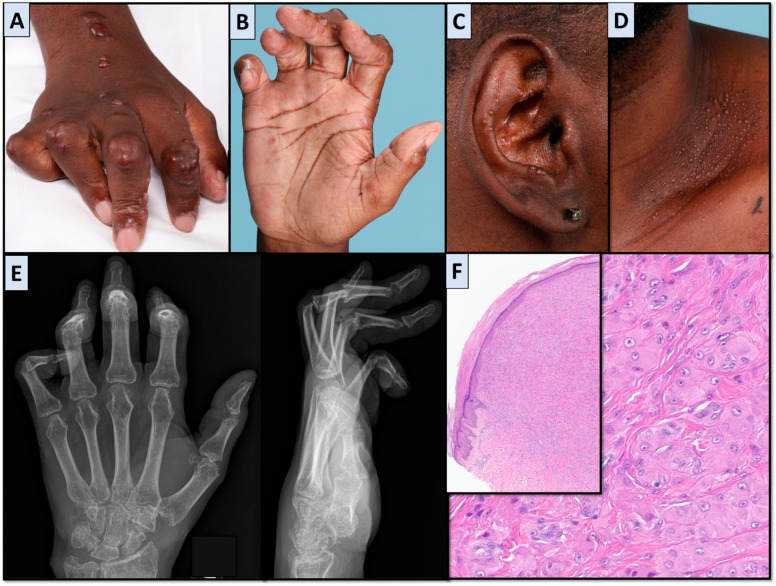
Multicentric reticulohistiocytosis images. Photographs demonstrating skin nodules affecting the (**A**) dorsal and (**B**) palmar right hand, (**C**) left ear, and (**D**) left neck, demonstrating skin nodules from Case 2. (**E**) Left-hand X-rays from Case 2. (**F**) Left neck nodule biopsy showing diffuse dermal infiltrate comprised of sheets of oncocytic histiocytes and multinucleated giant cells with eosinophilic, “ground glass” cytoplasm from Case 2 (hematoxylin-eosin, original magnification ×4 by digital scan in the inset, ×40 by digital scan in the large square).

MRH is a non-Langerhans cell histiocytosis characterized by symmetric polyarthritis associated with red or brown skin nodules. Extra-articular and non-dermatologic symptoms, including constitutional, pulmonary, cardiac, and mucosal, have also been described. The disease tends to occur around age 40 in white individuals with a noted female predominance. ESR and CRP have been noted to be elevated in 50% of cases. Autoantibody testing is usually unremarkable. It has an association with systemic autoimmune diseases, including rheumatoid arthritis and systemic lupus erythematosus in 15% of cases, as well as hematologic and solid malignancies in 25% of cases. Spontaneous remission within 10 years has been reported [7]. Hyperlipidemia and hypercholesterolemia have also been inconsistently appreciated [6]. Histology of skin and synovium demonstrates variable inflammatory infiltrate characteristically comprised of giant cells and mononuclear histiocytes with eosinophilic cytoplasm [6]. Similar to FR, classification or diagnostic criteria and guideline-based treatments are not available to help guide management. Methotrexate and steroids have been used to treat arthritis, while other therapies, including cyclophosphamide and chlorambucil, have been successful in treating dermatologic manifestations. Bisphosphonates have also been useful, with reported improvements in both skin and joint symptoms [6,7]. A recent review of the utility of tumor necrosis factor (TNF) inhibitors in the treatment of MRH noted several case reports showing improvement, for which this class of medications has been suggested as a potential steroid-sparing agent for refractory cases. This included 17 published cases of MRH with reported improvement in both cutaneous and articular symptoms, although not always both, with some reports describing benefit with an alternate TNF-inhibitor even if the first one was not as effective [8]. Characteristic MRH findings are shown in Figure 5. 

## 4. Disease Characteristics

In addition to the ability to differentiate these disease entities histologically, several arthritis characteristics have been compared. While RA and FR are correlated with juxta-articular osteopenia, MRH is not. MRH and FR demonstrate joint widening in comparison to RA, which demonstrates joint narrowing. All three diseases show marginal erosions, with FR being most likely to present with central erosions. MRH and FR involve DIP joints, the presence of which aids in differentiating them from RA [6,7]. Overall, MRH and FR are less responsive to available immunomodulatory therapies and lack management and treatment guidelines to guide their management. This is in the face of the potential for significant deformity and disability, given the erosive nature of the joint manifestations in these diseases (Table 1).

## 5. Other Considerations

In this review, FR and MRH are compared to RA in order to highlight an important diagnostic differential given how these mimics share the central manifestation of a polyarticular symmetric erosive inflammatory arthritis. Subcutaneous nodules, however, are not unique to these entities. Cutaneous nodules have been described in a broad differential spanning inflammatory conditions, infections, metabolic disorders, and malignancies. Examples of described nodule-associated entities include subcutaneous granuloma annulare, actinomyces, Farber disease, and amyloidosis [9]. To add further complexities to the differential, some cases may also present with arthralgias and frank inflammatory arthritis. Other historical, clinical, laboratory, and radiographic findings can help suggest consideration of one of these alternate disease processes [9].

## 6. Conclusions

Fibroblastic rheumatism, multicentric reticulohistiocytosis, and rheumatoid arthritis with nodules are three distinct conditions that can present with overlapping findings of cutaneous nodules and erosive polyarthritis. Skin biopsy of nodules for histopathology evaluation and consideration of the rarer diagnoses of MRH and FR in seronegative patients with arthritis presenting with fewer classic characteristics of RA may therefore be helpful in establishing the appropriate diagnosis. 

## Figures and Tables

**Table 1:** Comparison of nodule and arthritis findings in rheumatoid arthritis (RA), fibroblastic rheumatism (FR), and multicentric reticulohistiocytosis (MRH).

Condition	Joint Features				Nodule Features				
	Distribution	Erosions	Juxta-Articular Osteopenia	Joint Space	Distribution	Characteristics	Size	Histopathology	References
**RA**	Symmetric Spares DIP joints	Marginal	Present	Narrowed	Any region, classically extensor surfaces	Firm, nontender, sometimes mobile	20–50 mm	Palisading granulomatous dermatitis with central fibrin	[6,7,9]
**FR**	Symmetric, DIP joints involved	Marginal or central	Present	Widened	Extensor surfaces and para-articular	Flesh-colored to purplish; can be tender	2–20 mm (15 is median size)	Increased fibroblasts within a sclerotic dermis, few inflammatory cells	[6,7,9]
**MRH**	Symmetrical, DIP joints involved (marginal), joint widening	Marginal	Absent	Widened	Face, neck, hands; vermicular	Flesh-colored, red, or brown; can be pruritic	Millimeters (up to 20 mm)	Sheets of histiocytes and multinucleated giant cells with eosinophilic cytoplasm	[6,7,9]

## Data Availability

No new data were created or analyzed in this study.

## Abbreviations

The following abbreviations are used in this manuscript:

RA	Rheumatoid arthritis
FR	Fibroblastic rheumatism
MRH	Multicentric reticulohistiocytosis
MCP	Metacarpophalangeal
PIP	Proximal interphalangeal
CRP	C-reactive protein
ESR	Erythrocyte sedimentation rate
TNF	Tumor necrosis factor

## References

[1] Gabriel S.E. (2001). The epidemiology of rheumatoid arthritis. Rheum. Dis. Clin. N. Am..

[2] Tilstra J.S., Lienesch D.W. (2015). Rheumatoid Nodules. Dermatol. Clin..

[3] Chaouat Y., Aron-Brunetiere R., Faures B., Binet O., Ginet C., Aubart D. (1980). Une nouvelle entite: Le rhumatisme fibroblastique. Rev. Rhum. Mal. Osteoartic..

[4] Pieta A., Zioga A., Skalkou A., Venetsanopoulou A.I., Drosos A.A., Voulgari P.V. (2022). Fibroblastic rheumatism: An uncommon arthritis. A case-based review. Rheumatol. Int..

[5] Courties A., Guégan S., Miquel A., Duriez P., Berenbaum F., Sellam J. (2014). Fibroblastic rheumatism: Immunosuppressive therapy is not always required. Jt. Bone Spine.

[6] Trotta F., Colina M. (2012). Multicentric reticulohistiocytosis and fibroblastic rheumatism. Best Pract. Res. Clin. Rheumatol..

[7] Toz B., Büyükbabani N., İnanç M. (2016). Multicentric reticulohistiocytosis: Rheumatology perspective. Best Pract. Res. Clin. Rheumatol..

[8] Zhao H., Wu C., Wu M., Zhou Y., Zhu H., Li Y., You Y., Luo H., Wang L., Zuo X. (2016). Tumor necrosis factor antagonists in the treatment of multicentric reticulohistiocytosis: Current clinical evidence. Mol. Med. Rep..

[9] Evangelisto A., Werth V., Schumacher H.R. (2006). What is that nodule?: A diagnostic approach to evaluating subcutaneous and cutaneous nodules. J. Clin. Rheumatol..

